# Short-Term Ex Vivo Culture of CTCs from Advance Breast Cancer Patients: Clinical Implications

**DOI:** 10.3390/cancers13112668

**Published:** 2021-05-28

**Authors:** Nuria Carmona-Ule, Miriam González-Conde, Carmen Abuín, Juan F. Cueva, Patricia Palacios, Rafael López-López, Clotilde Costa, Ana Belén Dávila-Ibáñez

**Affiliations:** 1 Roche-Chus Joint Unit, Translational Medical Oncology Group, Oncomet, Health Research Institute of Santiago de Compostela (IDIS), Travesía da Choupana s/n, 15706 Santiago de Compostela, Spain; Nuria.carmona.ule@sergas.es (N.C.-U.); Miriam.gonzalez.conde@sergas.es (M.G.-C.); Carmen.abuin.redondo@sergas.es (C.A.); Rafael.lopez.lopez@sergas.es (R.L.-L.); 2 CIBERONC, Centro de Investigación Biomédica en Red Cáncer, 28029 Madrid, Spain; juan.fernando.cueva.Banuelos@sergas.es (J.F.C.); patricia.palacios.ozores@sergas.es (P.P.); 3 Translational Medical Oncology Group (Oncomet), Medical Oncology Department, University Clinical Hospital of Santiago de Compostela, 15706 Santiago de Compostela, Spain

**Keywords:** CTC, cell culture, liquid biopsy, breast cancer

## Abstract

**Simple Summary:**

Circulating tumor cells (CTCs) are responsible for metastasis, they represent tumor biology and have also predictive value for therapy monitoring and prognosis of metastatic breast cancer patients. In the blood, CTCs are found in low frequency and a small percentage of them survive. Therefore, achieving their expansion in vitro will allow performing characterization and functional analysis. In this work, we used growth factors and Nanoemulsions to support CTCs culture. We have seen that the CTCs subpopulation capable of ex vivo expanding presented mesenchymal and stem characteristics and loss of epithelial markers. Besides, CTC culture predicted progression-free survival.

**Abstract:**

Background: Circulating tumor cells (CTC) have relevance as prognostic markers in breast cancer. However, the functional properties of CTCs or their molecular characterization have not been well-studied. Experimental models indicate that only a few cells can survive in the circulation and eventually metastasize. Thus, it is essential to identify these surviving cells capable of forming such metastases. Methods: We isolated viable CTCs from 50 peripheral blood samples obtained from 35 patients with advanced metastatic breast cancer using RosetteSep^TM^ for ex vivo culture. The CTCs were seeded and monitored on plates under low adherence conditions and with media supplemented with growth factors and Nanoemulsions. Phenotypic analysis was performed by immunofluorescence and gene expression analysis using RT-PCR and CTCs counting by the Cellsearch^®^ system. Results: We found that in 75% of samples the CTC cultures lasted more than 23 days, predicting a shorter Progression-Free Survival in these patients, independently of having ≥5 CTC by Cellsearch^®^. We also observed that CTCs before and after culture showed a different gene expression profile. Conclusions: the cultivability of CTCs is a predictive factor. Furthermore, the subset of cells capable of growing ex vivo show stem or mesenchymal features and may represent the CTC population with metastatic potential in vivo.

## 1. Introduction

During 2020, breast cancer was the most commonly diagnosed cancer, with an estimated 2.3 million new cases [1]. Metastasis is the leading cause of death in breast cancer patients, mainly conditioned by resistance to therapy and tumor heterogeneity [2]. The circulating tumor cells (CTCs) are those cells that abandon the primary tumor and circulate through the vascular system to eventually colonize distant organs and originate the formation of metastasis [3]. High CTC numbers have been linked with worse outcome in prostate, colon and breast cancer metastatic patients [4,5,6] and the increase in number over time is an indicative of therapy failure [7]. However, CTC count does not allow therapeutic intervention. Therefore, CTC expansion in vitro would permit their downstream analysis, and evaluate personalized cancer therapies and drug sensitivity testing [8,9]. Even though CTCs are a highly valuable source of tumor material, their molecular analysis is still challenging due to their rarity, as they are as infrequent as <20 CTCs from 10 mL of blood [10]. Additionally, most of the CTC population dies in the bloodstream due to shear stress or immune surveillance [11]. Isolated CTCs show reduced proliferation hindering their ex vivo expansion [12]. Thus, nowadays CTC growth ex vivo is still one of the main challenges in the field, mostly due to extreme CTC rarity, fragility, and phenotypic heterogeneity.

Despite its obvious complexity, different cultures of CTCs have already been established. Thus, the first CTCs primary culture was established in 2013 from advanced breast cancer patients using CTCs negative for the epithelial marker EpCAM [13]. Later, in 2015, Cayrefourcq et al. established different cultures and a permanent cell line from CTCs isolated from a metastatic colon cancer patient, who presented a high number of CTCs (≥300 CTC) [14]. Currently, it is established that for long-term CTC cultures success, a high number of CTCs is required [15]. Numerous attempts have been made to culture CTCs from patient blood samples, however, the success rates of in vitro CTC cultures is around 6–20% [16]. To date, only a few short-term CTCs cultures, from 3 to 14 days but also up to 40 days, have been reported for different cancers [14,17,18,19,20,21,22,23,24,25,26], and specifically for breast cancer [16,27,28]. Nonetheless, the establishment of long-term CTC cultures has proven to be even more challenging for breast [13,28,29] and other tumor types [30,31,32], having much lower success.

Ex vivo tumor cell culture is usually performed in 2D cultures, however the cell morphology or cell-cell and spatial interactions are lost [33]. Since the importance of these interactions in different cellular functions as proliferation or cell differentiation has been demonstrated [34], 2D cultures show a considerable drawback. Furthermore, in 2D cultures nutrients or oxygen accessibility is unlimited [35], thus, this situation do not fully represents the setting in the tumor mass in vivo [36]. However, 3D cultures appears as a solution to overcome these limitations, simulating the environment of tumor cells, in which cell-cell and cell-environment interactions are maintained, as well as cell polarity and morphology [22,37]. Precisely, a non-adhesive substrate is employed to culture cancer stem-like cells (CSC) from primary tumors creating a multi-layered cell cluster [38,39]. Thus, for ex vivo CTC expansion, ultra-low attachment plates are frequently used [28,40,41,42]. Besides, hypoxic conditions (1–2% O_2_) can promote cellular reprogramming towards a CSC phenotype [43]. In vivo, tumor cells are supplied with growth factors, hormones, lipids and other metabolites from serum. Nonetheless, standard culture media are devoid of them, although they are essential for the CTC culture ex vivo [44]. Thus, the majority of protocols for CTC culture employ supplemented media to provide all the necessary nutrients for these rare cells [28,31]. 

In this regard, we have recently published a protocol that uses Nanoemulsions (NEs) formulated from lipids and fatty acid to promote the proliferation of breast cancer cell lines and CTCs from a mouse model derived from breast cancer cell line [45]. Our study was based on previous studies that reported how mitochondrial metabolism (including oxidation of glutamine and fatty acids) or the accumulation of lipids and free fatty acids is linked to invasion, aggressiveness, migration, and progression of breast cancer [46,47,48]. 

However, there is still the need of knowledge to know the optimal conditions that influence the success of CTC cultures and which are the specific populations capable of surviving in vitro. Therefore, to shed light on this field of research, we carried out a functional study of CTCs isolated from patients with metastatic breast cancer (mBC), placing them in culture and monitoring them over time. As a novelty, we used a culture protocol in which, together with growth factors, NEs (lipids and fatty acids) are added to the culture medium to stimulate the metabolism of CTCs.

## 2. Materials and Methods

### 2.1. Patients

This study was approved by the local ethics committee (Ethics Committee of Galicia approval reference number 2015/772) and all patients gave written informed consent. All samples were anonymized and encoded before the analysis.

Inclusion criteria: women, advanced breast cancer, any subtype, age ≥ 18 years. Exclusion criteria: known diagnosis of psychiatric illness that prevents the patient from understanding and accepting the conditions of the study, current of malignant tumor in the last 5 years, except for basal cell carcinoma, squamous cell carcinoma or carcinoma in situ of the cervix properly treated. The patient’s characteristics are described in Section 3.1 and Table 1. 

### 2.2. CTC Isolation and Ex Vivo Culture

Two EDTA-coated vacutainer tubes (Becton Dickinson, Franklin Lakes, NJ, USA) of 7.5 mL of peripheral blood were collected per patient at different time points. Blood samples were processed within two hours after withdrawal. CTC isolation was performed using the label-independent antibody cocktail RosetteSep™ CTC Enrichment Cocktail Containing Anti-CD56 (STEMCELL Technologies, Vancouver, BC, Canada). Enriched cells isolated with RosetteSep™ from the 1st EDTA tube were used for CTC culture as described below. In some random samples, the 2nd EDTA tube was processed for CTC enrichment by RosetteSep™, placed in RNAlater^®^ Solution (Invitrogen, ThermoFisher Scientific, Schwerte, Germany) and kept at −80 °C until further gene expression analyses. Besides, the Peripheral Blood Mononuclear cells (PBMCs) of some patients were isolated from one 7.5 mL EDTA tube of peripheral blood by density gradient centrifugation protocol (Lymphoprep™, STEMCELL Technologies, Vancouver, BC, Canada) in SepMate™ tubes (STEMCELL Technologies, Vancouver, BC, Canada) according to manufacturer’s instructions, for ex vivo culture, RNAlater storage and subsequent gene expression analysis.

Cell pellet from the previous step was resuspended in 200 µL of supplemented MammoCult^TM^ Human Medium Kit (STEMCELL Technologies). This supplemented medium contained 0.4 µg/mL progesterone (Sigma-Aldrich, St. Louis, MO, USA), 0.4 µg/mL ß-estradiol (Sigma-Aldrich), 4 µg/mL heparin (STEMCELL Technologies, Vancouver, BC, Canada), 0.48 µg/mL hydrocortisone (STEMCELL Technologies, Vancouver, BC, Canada), 5% (*v*/*v*) UltraGRO^TM^ (AventaCell BioMedical Co., Atlanta, GA, USA), 4% (*v*/*v*) B-27 Supplement (50X) (Gibco, ThermoFisher Scientific, Schwerte, Germany), 20 ng/mL recombinant basic Fibroblast Growth Factor (bFGF) (Gibco, ThermoFisher Scientific, Schwerte, Germany), 20 ng/mL recombinant Epidermal Growth Factor (EGF) (Sino Biological, Beijing, China), and 1%(*v*/*v*) penicillin/streptomycin (Sigma-Aldrich, USA). The cell suspension was seeded initially in one well of an Ultra-Low-Attachment 96-well plate (Corning, Merck Group, Darmstadt, Germany) and cultured using hypoxic conditions (37 °C 1–2%O_2_) for 1 week. Cell cultures were supplemented with fresh medium every 2 days with minimal well disturbance to avoid cell loss. NEs were prepared as previously described [45], and were added to the media every two days at the same time as the fresh medium supplement. Before addition to the media, NEs were filtered using Acrodisc^®^ PFS Syringe filters (Sigma-Aldrich, USA) and mixed with cell medium (concentration ranges were within 6.7 × 10^8^ particle/mL to 2.14 × 10^9^ particle/mL). After 1 week of cell culture, cells were switched to standard cell culture conditions (37 °C 5% CO_2_). When 85% confluence was reached, growing cells were transferred to an ultra-low attachment 24-well plate (Corning Inc., Merck Group, Darmstadt, Germany) and subsequently to Ultra-low attachment 25 cm^2^ Flasks (Corning) at 90% of confluence. Cultures were maintained up to the entrance to the decline phase (live cells declined as cell death predominated in the culture). Samples for immunofluorescence and RNA isolation were taken between 10 and 15 days after seeding.

### 2.3. CTC Enumeration

In 34 samples, one 10 mL CellSave Preservative tube (Menarini-Silicon Biosystems Inc, Bologna, Italy) was collected in parallel to EDTA tubes and processed for CTC enumeration by the CellSearch^®^ System, using CellSearch^®^ Epithelial Circulating Tumor Cell Kit (Menarini, Silicon Biosystems Inc., Bologna, Italy). EpCAM-positive enriched cells were labelled with phycoerythrin-conjugated anti-cytokeratins (8, 18, and 19) antibodies, allophycocyanin-conjugated anti-CD45 antibodies, and 4,6-diamino-2-phenylindole (DAPI). The CellTracks Analyzer (Menarini-Silicon Biosystems Inc., Bologna, Italy) was used to acquire digital images of the 3 different fluorescent dyes, which were reviewed by trained operators to determine the CTCs count.

### 2.4. Immunofluorescence Staining, Fluorescence Microscopy and Confocal Microscopy Analysis

Cells from 3D culture were washed with dPBS (Lonza, Basel, Switzerland) (centrifugation at 500× *g*, 5 min, 20 °C and fixed with cold 4% paraformaldehyde (PFA) (ThermoFisher, Schwerte, Germany) for 15 min. Afterward, cell solutions were immunostained using a combination of epithelial antibodies: Cytokeratin Pan Antibody (1:50, AlexaFluor-488 anti-human, Clone AE1/AE3, eBioscience-Invitrogen), EpCAM Antibody (1:25, AlexaFluor-488 anti-human, Clone 9C4, Biolegend), E-Cadh Antibody (1:25, FITC anti-human CD324, Clone 67A4, Biolegend); Vimentin Antibody (1:200, Alexa^®^-647 anti-human, Clone D21H3, CellSignaling Technology), and CD45 Antibody (1:30, PE anti-human, Clone MEM-28, ExBio). NucBlue^TM^ (Hoechst 33342 dye, ThermoFisher) was used to counterstain nuclei. InsidePerm Buffer (Inside Stain Kit, Miltenyi Biotec, Bergisch Gladbach, Germany) was used as antibody diluent.

Stained solutions were examined by (i) confocal microscope (Leica TSC SP5 X, Leica Microsystems, Wetzlar, Germany) and (ii) fluorescence microscope (Leica DMi8 automated Microscope, Leica Microsystems). In both microscopes, 63× oil immersion objective was used. Images were used for the morphometric analysis using ImageJ software [49], following previously described parameters [50]. 

### 2.5. RNA Extraction and Gene Expression Analysis

AllPrep DNA/RNA Mini Kit (Qiagen, Hilden, Germany) was used for RNA extraction (CTCs and PBMCs), following the manufacturer’s protocol. An amount of 11 µL of total RNA were retrotranscribed into cDNA with SuperScript III (ThermoFisher Scientific, Schwerte, Germany). Samples were preamplified with Taqman Preamp Master Mix (ThermoFisher Scientific, Schwerte, Germany). Gene expression analysis for a custom panel of 20 genes (Table S1) was performed with probes and Master Mix TaqMan (Applied Biosystems^®^, Foster City, CA, USA) on a LightCycler 480 II (Roche Diagnostics, Basel, Switzerland). The relative expression was calculated considering B2M as a reference gene. To clarify the visualization of the heatmap, relative expression was codified from 0 to 7 points, 0 being no gene amplification. 

### 2.6. Statistical Analysis

Statistical analysis was performed using GraphPad Prism 6.01 software (GraphPad Software Inc., La Jolla, CA, USA) and R Studio Version R-3.6.3. ClustVis tool was used for heatmap and PCA performance. Fisher exact test and Chi-square test were used for association analysis. We used the Wilcoxon signed-rank test for media comparisons (CTC enumeration between groups and gene expression comparison before and after culture using paired test). Progression-free survival (PFS) and overall survival (OS) were visualized using Kaplan-Meier plots and tested by the log-rank test and by univariate and multivariate Cox proportional hazards models. Progression was calculated from the moment the sample was taken until disease progression was detected, either by biochemistry or by imaging. Only *p* values < 0.05 were considered statistically significant.

## 3. Results

### 3.1. Patient’s and Samples Characteristics

A total of 50 samples from 35 metastatic Breast Cancer (mBC) patients were included in this study. Patients were enrolled from October 2017 to December 2020 and the end of follow-up was February 2021. Patient characteristics are shown in Table 1. The median age was 57.14 (range, 32–82 years). The molecular subtypes were Her2 enriched (*n* = 4), Luminal (*n* = 22) and Triple-negative (*n* = 9). All patients were stage IV and 88.57% had ≥2 metastatic locations. Additionally, 23 out of 35 had bone and visceral metastasis (65.71%), 3 out of 35 (5.57%) had only bone metastasis while 9 out of 35 (25.71%) presented visceral metastasis alone. Median PFS and OS were 6.3 and 10 months, respectively, considering the time of sample collection as the starting point. Regarding longitudinal samples, they were collected at different time points through the disease. Thus, 32 samples out of 50 (64%) were collected after the start of therapy. The remaining 36% were collected at baseline (diagnosis of metastasis). Of the treated patient samples, 14 were first-line and 18 had two or more lines of treatment (Figure S1). The treatments corresponding to the 35 patients were chemotherapy (74.29%), endocrine therapy plus CDK4/6 inhibitors (17.14%) or endocrine therapy alone. 

### 3.2. CTC Short-Term Culture from Metastatic Breast Cancer Patients

#### 3.2.1. CTC Culture Characterization

**Figure 1:**
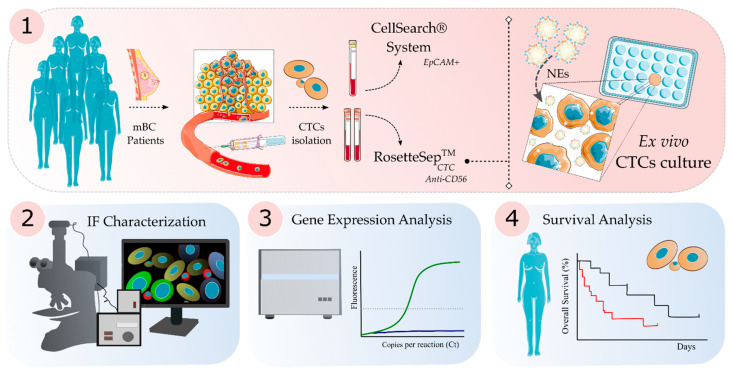
Schematic workflow of Circulating Tumor Cells (CTCs) samples isolation, culture and analyses. (1) Methodology followed for the isolation and ex vivo cell culture of CTCs from metastatic Breast Cancer (mBC) patients. Cells were isolated using: CellSearch^®^ System (determination of EpCAM^+^ cells) and RosetteSep^TM^ (enrichment of CTCs for cell culture). Cells were cultured under ultra-low attachment plates to support 3D culture and every two days cells were supplemented with fresh supplemented medium and Nanoemulsions (NEs). (2) Immunofluorescence characterization by confocal microscopy and fluorescence microscopy of cell cultures. (3) Gene expression analysis was performed using paired samples before and after cell culture. (4) Survival analysis was carried out to determine the relation between time (days) of culture or presence of Red Blood Cells (RBCs) as a predictive factor in the patient’s outcome.

CTCs isolated using a negative enrichment approach (Rosettesep^TM^), were plated under non-adherent conditions, in MammoCult media supplemented with growth factors plus NEs and under hypoxic conditions (for the first seven days) (see M&M). Figure 1 presents the schematic followed workflow in this study. Of the 50 initial samples, the follow-up was carried out in 48 of them, with a mean time of 56.35 days (range, 8–291 days). The criteria to consider the endpoint of the culture were cell density reduction or the presence of apoptotic cells. Some previous publications of short-term culture described maintenance of CTCs cultures ex vivo between 3 and 14 days [28,31]. Considering the median progression time (100 days) and the days of CTCs in culture, we performed an ROC curve analysis for these samples. The best threshold able to discriminate both groups was 23.5 days (AUC = 0.65). Therefore, we have considered the cut-off of 23 days as a successful CTC culture. Using this criterion, short-term cultures were successfully propagated in 36 samples (75%). In six of them, the follow-up was stopped due to culture contamination. Those samples in which this occurred before day 23 (*n* = 2) were considered as failure culture. 

**Figure 2:**
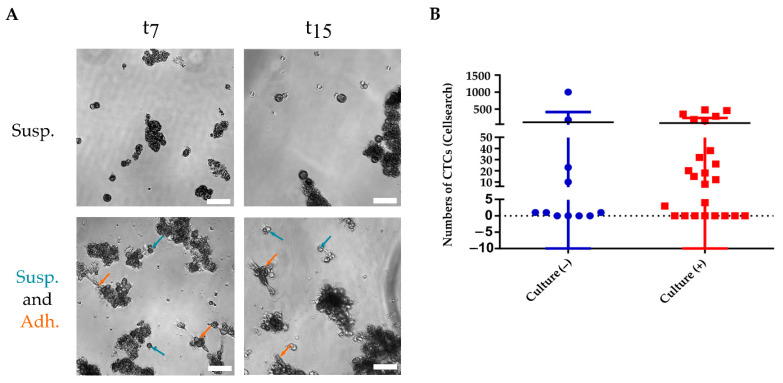
(**A**) Representative image of cultured Circulating Tumor Cells (CTCs) in ultra-low attachment 96-well plates at 7 and 15 days (t7, t15), left and right panel, respectively. Two growth behaviors were observed: (i) cells that grow in suspension (Susp.) (upper panel); (ii) cells growing in suspension (Susp.) co-existing with adherent cells (Adh.) (bottompanel). Blue arrows represent cells in suspension while orange arrows point to cells growing in adherence. Both samples had not receive treatment (basal). The scales bar represent 75 µm. (**B**) Number of CTCs measured by CellSearch^®^ with respect to their ability to establish a successful culture in 34 metastatic breast cancer samples. Negative culture, in blue (*n* = 11) and positive culture in red (*n* = 25).

Since cells were grown on ultra-low attachment plates, the majority tended to grow in suspension. Interestingly, some samples presented cells growing both in suspension and adherence as is observed in Figure 2A, where images were taken at day 7 (once cells were removed from hypoxia) and day 15 (when the cells were collected for characterization).

In 34 samples from 28 patients, paired CTC enumeration was performed by the Cellsearch^®^ system at the time of the start of culture. The Cellsearch^®^ system isolates CTCs with an epithelial phenotype since it bases the isolation on the epithelial adhesion molecule (EpCAM), (Figure S2). Fifty-three per cent of the samples showed ≥5 CTCs (range 0–1000) although only four of them had ≥350 CTCs. No association was observed between culture successes and having ≥5 CTC (pre-established cut-off in mBC). The highest CTCs counts were observed in the successful or positive culture group (Figure 2B), however, there were no significant differences in the mean of CTCs numbers in both groups (Table S2). 

**Figure 3:**
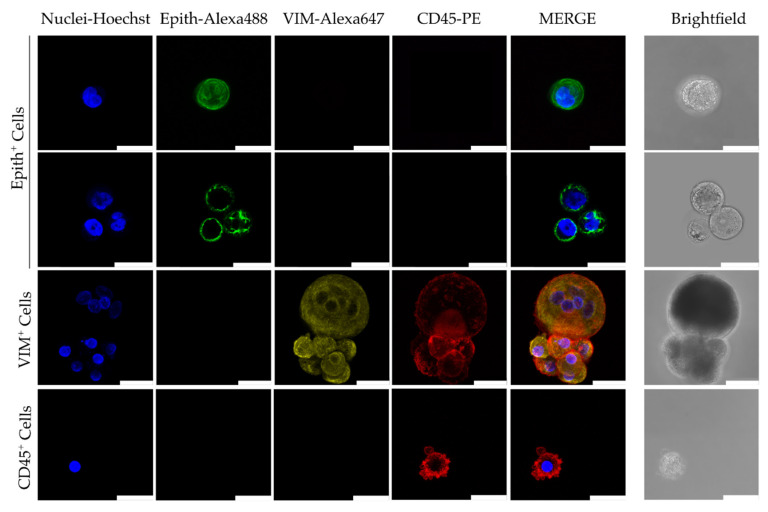
Representative images by confocal microscopy of Immunofluorescence characterization of Circulating Tumor Cells (CTCs) after culture. Immunofluorescent staining was performed using a combination of anti-human Epithelial markers (Epith: EpCAM, E-Cadh, and PanCK) (in green), anti-Vimentin (VIM, in yellow), and anti-CD45 (in red). Scale bar represents 25 µm.

After ~2 weeks in culture, it was performed a phenotypic characterization in some random samples. For that, we carried out a triple immunofluorescence assay for epithelial (E-Cadh, EpCAM, and PanCK), (Figure 3), mesenchymal (Vimentin) and white blood cells markers (CD45). Epithelial features were observed in a low percentage of samples (18.18%) (*n* = 22) while 47% showed vimentin-positive cells (*n* = 17), or no marker but nucleus in scarce samples. Of the samples analyzed by immunofluorescence, only four did not result in a culture-positive although none of them showed positive marker expression by immunofluorescence. We also detected CD45+ cells co-inhabiting in the culture in the majority of the samples. In another vein, Cellsearch^®^ data (before culture) did not associate with the expression of epithelial markers after culture. 

Since some cells did not meet the standardized CTC criteria (CK+; CD45–; DAPI+), we performed a morphometric analysis of some of the immunofluorescence images (*n* = 82 from 18 patients). Although there are no standardized data, different research groups have reported that CTCs exhibit a larger nucleus size (>9 µm) and nucleus/cytoplasm ratio (>0.8) [50]. We observed that the nucleus from cells with epithelial markers, Vimentin or absence of any marker are larger than those CD45-positive cells (mean 12.97 vs. 10.27 µm, *p* = 0.008). Furthermore, considering both nucleus size (>9 µm) and the nucleus/cytoplasm ratio >0.8, CD45-negative cells meet the criterion preferentially (*p* = 0.02).

In 70.9% of samples, was observed contamination with RBCs in the culture that eventually disappeared. The presence of RBCs did not associate with culture success nor with CTC account. Besides, the success of the culture did not show an association with the molecular subtype, the age of the patient, the therapy received or the visit in which the sample was collected (before or after therapy initiation; first line or ≥2 lines of treatment). 

#### 3.2.2. Gene Expression Analysis of Paired Samples before and after Culture

Next, we performed a gene expression analysis for a panel of 20 genes before and after culture (~2 weeks) in a small set of samples. All of them were in culture for more than 23 days. The genes selected included epithelial (*KRT5*, *CDH1*, *EpCAM)*, mensenchymal (*VIM*, *SNAI1*, *TWIST*), stem (*ALDH1A1*, *PROM1*), breast specific (*ESR1*, *PALB2*) and other genes related to cell cycle or other cancer pathways (*CCND1*, *CTNNB1*, *Ki67*, etc.). 

**Figure 4:**
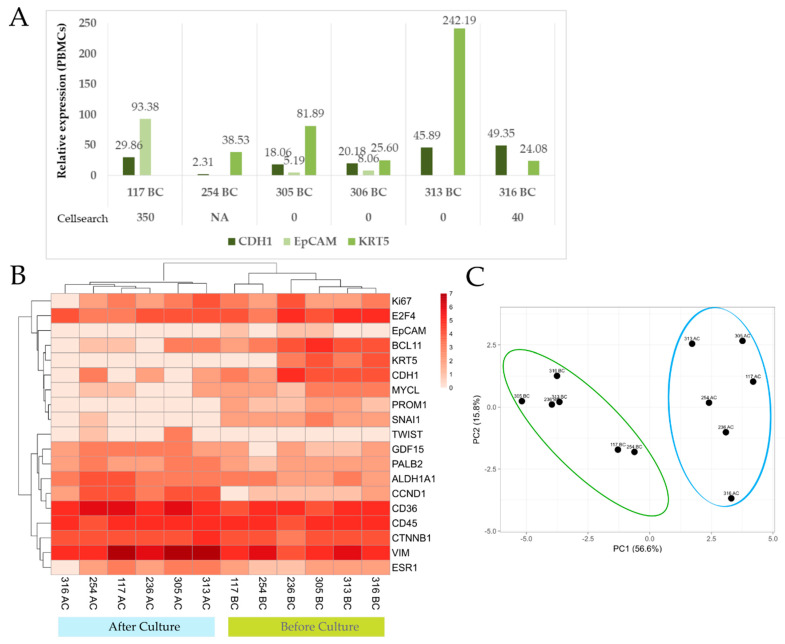
CTC gene expression analysis of six samples. Of these samples, four of them were not included in the previous immunofluorescence characterization. Five samples were collected after therapy initiation. (**A**) Relative gene expression for epithelial markers (E-Cadh, EpCAM and KRT5) of CTC-fraction isolated by Rosettesep and normalized with paired Peripheral Blood Mononuclear Cells (PBMCs) (before culture). Comparison with Cellsearch^®^ data is shown. (**B**) Clustered heatmap depicting CTCs gene expression levels of the listed genes. Light red indicates no expression. Relative gene expression (to B2M) was ranged and coded from 1 to 7 (dark red). (**C**) Principal component analysis using gene expression of the listed genes (see Heatmap).

Remarkably, epithelial markers were detected preferentially in the CTCs freshly isolated from the blood, which have not been cultured (Figure 4A,B). CTCs before and after culture shown a differential gene expression pattern as depicted in the Principal Component Analysis diagram and hierarchical clustering, which grouped the samples based on whether they had been in culture or not (Figure 4B,C). Breast-specific markers as *PALB2* or *ESR1* were expressed independently of the culture state, although both genes showed a higher relative expression in CTCs from cultured samples (*p* = 0.03 and 0.06, respectively) suggesting that mammary tumor cells were proliferating in the culture. The mesenchymal gene *VIM* was found in all the samples before and after culture, *TWIST* expression appeared only after culture in two out of six samples while *SNAI1* is lost. VIM was overexpressed in four out of six samples after culture. One of the samples, after culture, showed less VIM expression and an increase in *CDH1,* which indicates a maintenance of epithelial features. *CD45* relative expression showed a slight drop after culture. 

Taking into account that the isolation method used is not characterized by its purity, non-tumor cells may be contaminating the culture. This can influence the gene expression analysis of cells after culture, analyzed as a pool. For this reason, in four of the six samples, we analyzed the expression of PBMCs before and after culture (cultured under the same conditions that the CTC- enriched fraction).

CTCs after culture exhibited a differential expression profile, ruling out that gene expression is influenced by non-specific cells that accompany the culture of CTCs (Figure S3A,B).

In addition, other EMT-related genes were analyzed. Thereby, *CTNNB1* and *GDF15* showed higher relative gene expression after culture (*p* = 0.03 and 0.06, respectively). This is in agreement with previous findings in which mesenchymal markers were mainly observed after culture. Additionally, *CCND1* showed an increase but was not significant. Notably, the expression of *CD36* and *ALDH1A1* was detected in all CTC samples (before and after culture) and it is significantly elevated in cultured samples (*p* = 0.03) (Figure S3C,D). Since *CD36* is a marker associated with certain cells of the myeloid lineage we checked whether *CD36* expression could come from PBMCs present in the culture. For that, we compared the relative expression of CTC and PBMCs after and before culture. We observed a notable overexpression in CTCs after culture (*p* = 0.002) while PBMCs after culture just showed a slight increase (*p* = 0.64) (Figure S3E), reinforcing that the expression of *CD36* comes from CTCs.

In summary, CTCs after culture showed mesenchymal and stem cell features together with breast-specific markers. Besides, their genetic profile differs from culture-paired PBMCs, reinforcing the tumoral origin of culture cells. 

### 3.3. CTC Cultivability for More than 23 Days Predicts the Patient’s Progression

**Figure 5:**
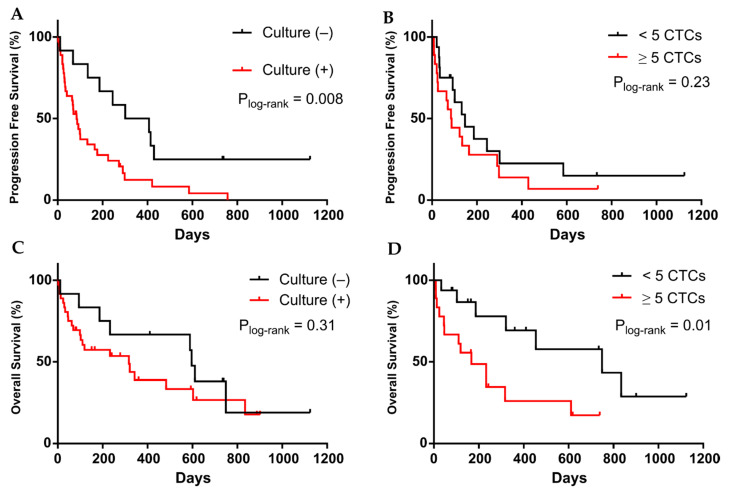
Kaplan–Meier plots for Progression free Survival (PFS) and Overall Survival (OS) for: cultivability (Culture negative (black) or positive (red)) (**A**,**C**) and CTCs enumeration by Cellsearch^®^ system (≥5 CTCs (red) or <5 CTCs (black) (**B**,**D**). *p*-values were calculated using the log-rank test.

Last, considering the cultivability (positive or negative based on being >23 days in culture), we performed survival analysis to determine if the culture time could predict the outcome of the patients. Patients whose samples originated cultures that lasted more than 23 days, considered positive cultures, showed shorter times to progression (*p* = 0.008, log-rank test, 354 vs. 84 days; *n* = 48), (Figure 5A). If we only consider those samples with paired Cellsearch^®^ data (Figure S1), culture-positive also predicted shorter PFS (*p* = 0.002, log-rank test, 365 vs. 84 days; *n *= 32), (Figure S4A). On contrary, having ≥5 CTCs (measured by Cellsearch^®^) did not predict patients PFS (*p* = 0.2, log-rank test, 147 vs. 85.8 days; *n* = 32), (Figure 5B). Besides, a positive culture was also predictive (*p* = 0.05, log-rank test, *n* = 34), (Figure S4B) when only one sample per patient was considered (Figure S1). The culture success was 75% and 76.47%, respectively, for these last samples’ cohorts.

Regarding OS, culture positivity was not a predictive factor in these samples (*p* = 0.3, log-rank test, 596 vs. 317 days; *n* = 48), (Figure 5C), although the mean OS times of both groups were significantly different (592.5 vs. 158 days, *p* = 0.01). Contrary to PFS analysis, having ≥5 CTCs was linked with shorter OS (*p* = 0.01, log-rank test, 749 vs. 167 days; *n* = 34), (Figure 5D). 

**Figure 6:**
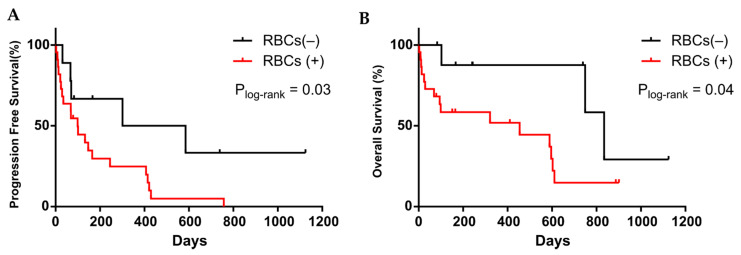
Kaplan–Meier plots for PFS (**A**) and OS (**B**) for presence of RBCs (red) or absence (black) in the culture. *p*-values were calculated using the log-rank test.

Interestingly, we found that the presence of RBCs in the culture was linked to a worse patient outcome (*p* = 0.03, log-rank test, 443 vs. 99 days for PFS and *p* = 0.04, log-rank test, 834 vs. 454 for OS, *n* = 31), (Figure 6A,B). When it was considered only those samples with Cellsearch^®^ data or just one sample per patient, the presence of RBCs in culture was also predictive for PFS (*p* = 0.01 and 0.05 respectively), (Figure S4C,D). Additionally, the amount of RBCs in culture tended to correlate with shorter both PFS and OS (*p* = 0.05 and 0.03, respectively), (Figure S4E–G). The same trend was also observed with the amount of RBCs in the culture plate in the aforementioned samples’ cohorts. 

In the multiple regression analysis, CTC maintenance in culture for more than 23 days and the presence of RBCs in the sample were found to be predictive factors for PFS (*p* < 0.05) after adjustment for other clinical variables (age, molecular subtype or treatment received (type and number of lines of therapy)).

## 4. Discussion

There are multiple publications where the value of CTCs for patient follow-up has been reported, especially concerning the enumeration of CTCs [51,52]. In recent years, progress has also been made in the detection of mutations and phenotypes in CTCs that can be clinically relevant [53,54]. However, there is still a gap to know if CTCs can provide information for routinely clinical decision making, especially for precision medicine. Comprehensive characterization of CTCs in transcriptomic, genomic, and functional terms is difficult mainly due to their low frequency in blood, heterogeneity, poor survivability and challenging methods of CTC isolation. Having CTC lines will be a huge advance to deepen our knowledge in the biology of CTCs and will permit researchers to perform CTC functional studies, allowing the characterization of those tumor cells responsible for metastasis [55]. Currently, the success to generate stable lines of CTCs is subjected to a high tumor burden [56], which represents a fatal clinical condition for the patient. Thus, CTC culture is one of the present challenges in the liquid biopsy field. To date, few CTCs cell lines have been obtained [11,26,27,28,29,30]. 

In this work, we have carried out a functional characterization of CTCs, studying their ability to grow in culture, to decipher which are the features that boost their culturability. For that, we choose a negative isolation CTCs enrichment approach, to guarantee minimal cell damage and to obtain a heterogeneous CTC population. The CTCs were cultured under ultra-low attachment conditions using a specific protocol, which includes the addition of NEs as previously described by our group [45]. The addition of biocompatible NEs composed of lipid and fatty acids to support CTC cultures, improving their proliferation capacity, has not been reported to date. The advantage of NEs promoting cell proliferation is supported since it is known that the accumulation of intracellular lipids can contribute to tumor progression and metastasis [57,58,59]. Thus, the availability of extracellular lipids and fatty acids in the medium might help to activate the metabolism of cultured cancer cells. By employing the aforementioned protocol, we observed that CTCs can grow ex vivo for weeks (mean 8 weeks). It was observed a positive culture in 75% of the analyzed samples, implying more than 23 days with proliferative and survival features, which is a higher percentage than previously published works which reported 13 or 28% of success [60,61]. Cultured CTCs showed a specific phenotype with low epithelial markers and the presence of mesenchymal markers such as vimentin. This is in agreement with different published studies that indicate that after 14 days in culture, CTCs lost their epithelial features [27,62] and exhibited mesenchymal marker [25,63]. Thereby, Khoo et al. maintained CTC cultures for 2–8 weeks and observed that CTC in culture over time presented more mesenchymal characteristics, with an increase in vimentin and fascin staining and almost a complete loss of epithelial characteristics. They also observed that some CK+ cells presented vimentin expression, suggesting the presence of intermediate phenotypes [27]. 

Previous works have reported how CTCs in the blood circulation suffered from fluid shear stress, promoting EMT phenotype and maintained pre-existing mesenchymal cells [64]. Hence, most tumor cells released into circulation can be eliminated by fluid shear stress and if so, those CTCs capable of surviving and resisting fluid shear stress should be the subpopulations capable of generating metastatic tumors in vivo or cultures of CTCs in vitro. Accordingly, due to the CTCs culture conditions, as non-adherent conditions, we are boosting the survival of CTCs stem-like phenotype.

Interestingly, in our cultures, we found expression of *GDF15* and *CTNNB1*, both genes related to EMT and stemness. On one hand, *GDF15*, a member of the TGF-β superfamily, has been linked with macrophages, adipocytes, and several carcinomas including breast cancer. *GDF15* plays a dual role in the evolution of cancer [65]. Thus, under normal physiological conditions, it inhibits early tumor promotion. However, its abnormal expression in advanced cancers causes proliferation, angiogenesis, invasion, metastasis, drug resistance, stemness or immune escape [66]. On the other hand, *CTNNB1*, which encodes beta-catenin, has been linked to the EMT process, CSC pluripotency, and cancer signaling [67]. Besides, it has been seen that overexpression of *CTNNB1* in CTCs has prognostic value in both cancers, melanoma [68] and pancreatic [69]. Additionally, *ALDH1A1*, a stem cell marker linked with mammary stemness [63] was also increased after culture. This is in agreement with Zhang and colleagues who established long-term culture from CTCs ALDH1A1(+); EpCAM(−) in breast cancer suggesting that this CTC population was able to form brain metastasis [13]. It has been reported that EMT induction leads to stem markers expression and increased tumorigenic capacity in mouse models [70,71] and that the EMT program led to a spectrum of cell states with mixed epithelial and mesenchymal characteristics. Importantly, only those cells with the intermediate phenotype and stem feature associate with metastatic spread [72]. This agrees with our data since we saw that the cells that survive in culture show mainly stem or mesenchymal features.

Importantly, genes related to breast cancer, such as *ESR1* or *PALB2* were observed in all the analyzed samples, before and after culture. After culture, an increase in their relative expression was detected, pointing out that the subset of CTCs growing in culture is mainly of mammary origin. Besides, *PALB2* expression in CTCs was already described and linked with worse outcome in advanced breast cancer patients by our group [73]. Furthermore, we have seen that cells in culture with epithelial characteristics or absence of CD45 meet the morphometric criteria of CTC described by other authors [50], reinforcing the tumor origin of cells in culture. In recent years, the standard CTC condition has included morphological criteria [74,75,76,77,78]. It is known that there are distinct subpopulations of CTCs with differential expressions and morphological data. Besides, the conditions in which these parameters are obtained (before or after suspension culture; suspension or adherence; immunofluorescence or immunocytochemistry assay, CTC-isolation approach) can lead to a variety of results. Therefore, there is no universal criterion, although it is clear that cytometric analyses discriminate CTCs from blood cells (monocytes, granulocytes or tumor-associated macrophages) [79].

After culture, we observed a lower expression of CD45 compared with their counterparts before culture which may indicate that the culture favors tumor cells growth. CTCs before culture seem to show higher contamination of CD45+ cells, as supported by the hierarchical clustering which groups these samples interspersed with PBMCs while CTCs after culture sited together. Even though, in the majority of cultured samples, we detect associated CD45+ cells, similarly to other related publications [27] which reported the benefit of CD45+ (leukocytes) to support CTCs survival [80]. It is well known that the chosen CTC-enrichment approach has some drawbacks as the inefficiency in eliminating leukocyte contamination, resulting in low purity [81]. Hence, finding CD45+ cells in culture initially can be thought as a sign of impurity. However, these cells could be supporting the survival of CTCs ex vivo. It has been previously reported how CTCs associated with leukocytes in circulation promotes metastasis [80]. Recently, Xiao et al. have described that cells concomitant with CD45+ cell in short-term culture (30 days) of mBC samples displayed higher growth potential [16]. It would be interesting to characterize in-depth the population of contaminating CD45+ cells to know if they present specific characteristics and to determine if their nature is indeed capable of conditioning the achievement of the culture. However, we have no data regarding concomitant expression of stem cell markers and CD45 after culture since gene expression analysis was performed in a pool of CTCs. It has also been published that the fusion of myeloid cells with CSC promotes metastasis in lung cancer. These hybrid cells escape the immune system and are capable of colonizing distal organs [82]. This phenomenon could be favoring the presence of CD45+ cells in the CTC enriched samples, which would explain its contribution to culture success. Besides, in the same work, Aguirre and colleagues have seen that this fusion depends mainly on the expression of CD36 in CSCs since it is capable of adapting tumor cells to microenvironmental conditions, especially related to energy supply [82]. We have seen an overexpression of CD36 in the CTCs after culture. It has been previously suggested that the addition of lipids and other metabolites to culture media may help the ex vivo growth of tumor cells as serum does in vivo [44]. Besides, in CSC metabolic has been observed reprogramming [83] since they use mainly fatty acid oxidation and ketone bodies as the main source of energy production. In this work, for the first time, culture media was supplemented with NEs composed of oleic acid and lipids for CTC growth. Thus, the increase in the CD36 fatty acids transporter expression can be associated with the addition of oleic acid to the CTC cultures. Previous work showed that after exogenous addition of oleic acid to breast cancer samples, CD36 expression increased, followed by accumulation of cytoplasmic lipid droplets only in CD36-expressing cells [84]. These findings are in agreement with our previous work where we observed an accumulation of cytoplasmic lipid droplets in breast cancer cells after the addition of NEs, which lead to increased cell viability [45]. In addition, CD36 was already linked with metastatic potential in breast and melanoma cancer metastasis [85] and as a key element of survival in resistant breast cancer cells [86].

Hence, CTCs gene expression before and after culture exhibit a different profile, probably due to the survival of a specific subpopulation with mesenchymal and cancer stem features. The survival and proliferation advantage could have been acquired through evolution from the primary tumor. More interestingly, the ability of CTCs to grow ex vivo predicts patient outcome, in terms of PFS. Having biomarkers for PFS prediction can be very useful to evaluate the response to therapy in short times. Thus, the CTC population prone to survive and proliferate in vitro could reflect the malignancy of CTCs and the patient’s state. Previously, data from Khoo et al. suggested that cultured CTCs can be tools for early prediction of response to therapy. They observed less cell cluster formation in patients with longer treatments, although their data were not statistically significant, probably due to the low number of cases studied (*n* = 14) [27]. Unexpectedly, our data showed that having ≥5 CTCs was not related to PFS nor with successful cultivation. Only 53% of samples were positive for CTCs by Cellsearch^®^ (cut-off of ≥5 CTCs) compared with 75% of cultivability in this cohort. It should be noted that the enumeration of CTCs was performed using an epitope-dependent technology (Cellsearch^®^) that bases its isolation on EpCAM+ cells, missing stem/mesenchymal cells-like phenotype. For in vitro culture we use a negative enrichment approach that isolates all populations of CTC [33]. For this reason, the comparison of both systems might be biased and the lack of association may be precisely due to the different subpopulations that could be contributing to the cultivation and its success. Remarkably, the presence of RBCs, evaluated at the beginning of culture, is linked with a worse patient’s outcome although it does not influence the success of the culture. Unspecific RBCs isolated in the CTC-enriched fraction were mainly seen in patients with advanced disease, but not in patients in early stages or healthy donors (data not shown). It has been described that oncologic patients eventually display anemia and coagulation issues [87,88,89] but as far as we know, the causes are unknown. The non-specific isolation of RBCs in patients with breast cancer could reflect these imbalances in hematopoietic homeostasis due to bone marrow affectation or other alterations derived from the therapy or the general state of these patients. 

In this study, there is a limitation due to the lack of pheno- and genotypic characterization of samples. Through immunofluorescence, we can evaluate the co-expression of markers but we have limited the number of genes explored. This is something that we have tried to replace with the gene expression analysis, although it does not offer exhaustive information on how the co-expression of genes have taken place. It is necessary to take into account that once the cells are plated, the manipulation and removal of small amounts for analysis on many occasions conditions its culture continuity. Therefore, characterization was not carried out in each sample included in the study. Besides, CTCs can modify their expression as a consequence of culture conditions (low adherence (3D), addition of growth factors or even the isolation technology used) [27,62]. 

The short-term culture of CTCs, as well as the presence of RBCs, could be useful tools in the clinic, due to their predictive value of patient’s outcome. Since the results are rapid (on day 23 or immediately, respectively), those patients with positive cultures or the presence of RBCs may require more exhaustive follow-up. Furthermore, CTC culture could also be an intermediate step in the generation of CTC-derived xenograft (CDX) as previously described by our group [90]. Since CD36 is overexpressed in CTCs after culture, they may have a greater potential to generate CDX in vivo, taking into account the suggestion that CD36 mediates migration and invasion capacity in breast cancer [91]. However, CDX development shows certain limitations for a direct clinical application due to the long times required for xenographting [31,92]. Nevertheless, our in vitro CTC culture protocol is simple and less time-consuming compared to in vivo models. Thus, these cultures, in their proliferative phase, could be potentially used for drug-testing and molecular characterization for personalized oncology and is something to explore in the future.

## 5. Conclusions

In this work, we have described for the first time the use of NEs to support the cultivation of CTCs from patients. To our knowledge, this is also the first time that the cultivability of CTCs is a predictive factor in mBC. Interestingly, the samples included in this work represents real patients CTCs, adding more value to the study. The analysis of CTCs in culture has also allowed us to study the precursor cells, characterized by presenting mesenchymal and stem features. In addition to culture capacity, the presence of RBCs in culture is also of great relevance. Even so, the potential of CTC functional characterization is not fully understood, as the possibilities of short-term culture are not well-defined.

## Figures and Tables

**Table 1:** Clinic-pathologic characteristics of the cohort of metastatic breast cancer patients (*n* = 35).

Category	*n*	%
Age		
≤57	18	51.43
>57	17	48.57
Tumor stage		
IV	35	100
ER status		
Positive	21	60
Negative	12	34.29
PR status		
Positive	15	42.86
Negative	18	51.43
HER2 status		
Positive	8	22.86
Negative	26	74.29
Metastasis location		
Bone	26	74.29
Visceral	32	91.43
Bone & Viceral	23	65.71
Number of metastatic sites		
1	4	11.43
2	13	37.14
≥3	18	51.43
Therapy		
Chemotherapy	26	74.29
CDKi + ET	6	17.14
ET	2	5.71
Lines of therapy		
Basal	14	40
1 Line	7	20
≥2 Lines	14	40
Progression	31	88.57
Exitus	24	68.57

ER = Estrogen Receptor, PR = Progesterone Receptor, HER2 = Human Epidermal Growth Factor Receptor 2, CDKi = Cyclin Dependent Kinase inhibitor, and ET = Endocrine Therapy.

## Data Availability

The data presented in this study are available in this article and Supplementary Materials.

## Supplementary Materials

The following are available online at https://www.mdpi.com/article/10.3390/cancers13112668/s1, Figure S1: Diagram of the samples used in the analysis; Figure S2: Representative image of CTC (Cellsearch^®^); Figure S3: Gene expression analysis; Figure S4: Survival analysis of alternative samples cohorts. Table S1: Probes of the listed genes for Gene Expression Custom Panel; Table S2: Detailed description of Cellsearch CTC count and days in culture of CTCs.
