# Supplementary material for: Short-Term Ex Vivo Culture of CTCs from Advance Breast Cancer Patients: Clinical Implications
Source: Cancers (Basel). 2021 May 28;13(11):2668. doi: 10.3390/cancers13112668 (PMC8198105; doi:10.3390/cancers13112668)
Supplement: Supplementary file 1 [file cancers-13-02668-s001.zip › cancers-1204705-supplementary.pdf]

Supplementary Materials:

Short-Term Ex Vivo Culture of CTCs from Advance Breast Cancer Patients: Clinical Implications.

Nuria Carmona-Ule, Miriam González-Conde, Carmen Abuín, Juan Cueva, Patricia Palacios, Rafael López-López, Clotilde Costa and Ana Belén Dávila-Ibáñez

Table S1. Probes of the listed genes for Gene Expression Custom Panel.

TaqMan Probes	Reference
ALDH1A1	Hs00946916_m1
BCL11	Hs01093197_m1
CCND1	Hs00765553_m1
CD36	Hs00169627_m1
CD45	Hs04189704_m1
CDH1	Hs00170423_m1
CTNNB1	Hs00355049_m1
E2F4	Hs00608098_m1
EpCAM	Hs00158980_m1
ESR1	Hs01046816_m1
GDF15	Hs00171132_m1
Ki67	Hs00171132_m1
KRT5	Hs00361185_m1
MYCL	Hs00420495_m1
PALB2	Hs00226617_m1
PROM1	Hs01009257_m1
SNAI1	Hs00195591_m1
TWIST	Hs01675818_s1
VIM	Hs01675818_s1
B2M	Hs00187842_m1

Table S2. Detailed description of Cellsearch CTC count and days in culture of CTCs.

Patient´s Code	Treated	Cellsearch® Count	CultureDays	Culture	Phenotype	Subtype
#1_a	yes	0	24	+	NA	TN
#1_b	yes	1	21	-	EPI-	TN
#1_c	yes	0	44	+	DAPI+	TN
#2	no	455	85	+	NA	Luminal
#3	yes	0	51	+	NA	TN
#4	yes	3	24	+	NA	Luminal
#5	no	0	12	-	NA	Luminal
#6	no	38	25	+	NA	Luminal
#7	yes	4	24	+	NA	Luminal
#8	yes	1	16	-	NA	Luminal
#9	yes	288	25	+	NA	Luminal
#10	yes	350	85	+	DAPI+	Luminal
#11	no	1000	23	-	VIM-	HER2
#12	yes	0	20	-	NA	HER2
#13_a	no	10	9	-	EPI-	Luminal
#13_b	no	0	8	-	NA	Luminal
#14	yes	0	NA		NA	Luminal
#15_a	no	0	291	+	NA	TN
#15_b	yes	8	27	+	NA	TN
#16	no	25	NA		NA	TN
#17	no	15	172	+	VIM+; EPI-	Luminal

#18	yes	32	155	+	VIM-; EPI-	Luminal
#19	yes	20	151	+	NA	Luminal
#20	yes	159	69	+	NA	Luminal
#21	no	180	9	-	EPI-	TN
#22	yes	0	36	+	EPI-	TN
#23_a	no	12	91	+	VIM+;EPI-	TN
#23_b	yes	26	59	+	VIM+;EPI+	TN
#23_c	yes	0	44	+	DAPI+	TN
#24	yes	190	30	+	NA	Luminal
#25	yes	18	44	+	DAPI+	Luminal
#26	yes	477	43	+	VIM+;EPI+	Luminal
#27	yes	0	44	+	VIM+;EPI-	TN
#28	yes	0	40	+	VIM+;EPI-	Luminal

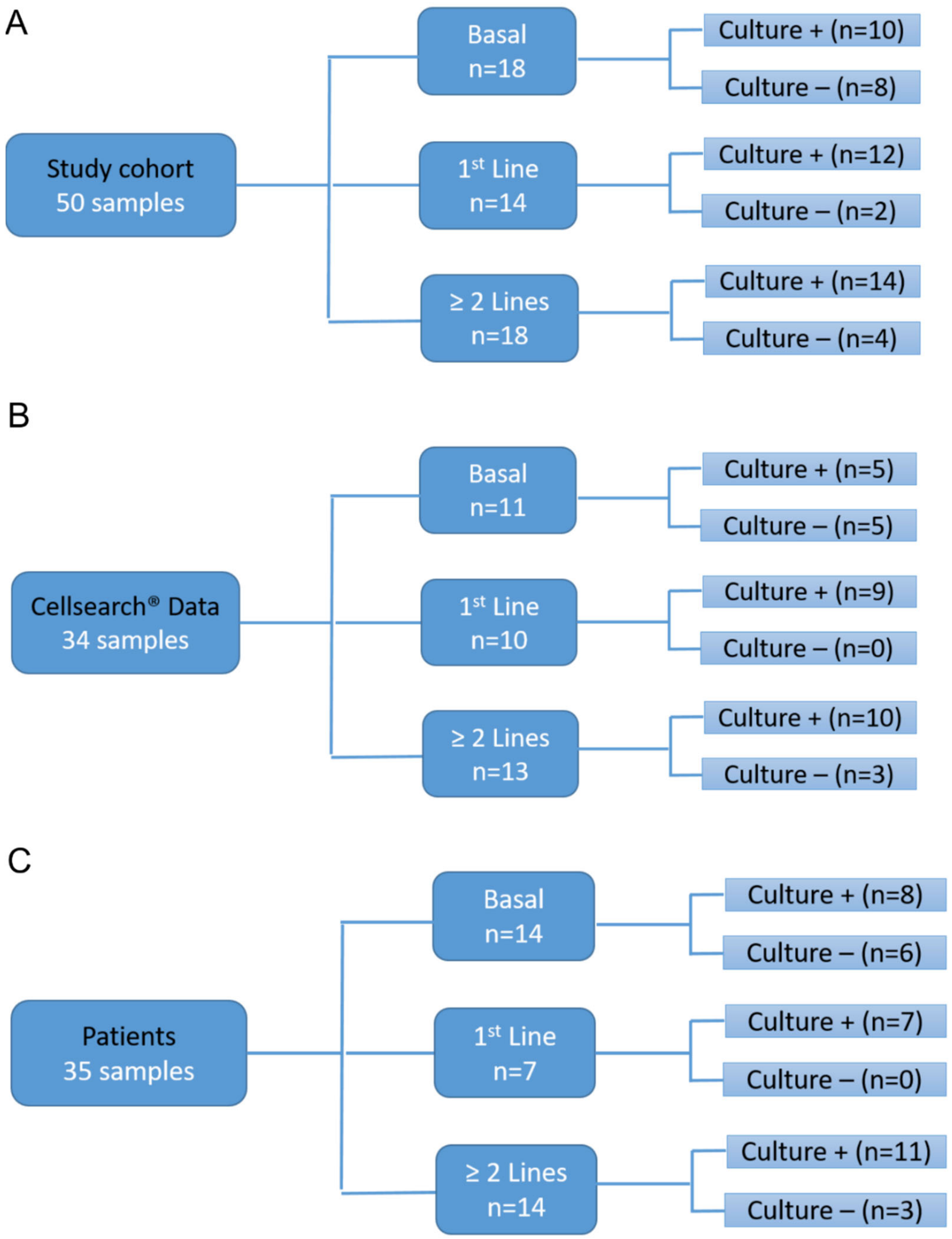

Figure S1. Diagram of the samples used in the analyses, which describes the number of samples included in each cohort, the treatment at the time of sample collection, and their distribution positive or negative culture. (A) The study cohort

includes 50 samples from 35 patients. (B) Those samples in which there are paired data of enumeration of CTCs by Cellsearch®. (C) Those samples in which only one sample per patient is considered. In patients with different samples collected, the basal sample was selected preferentially. In 9 patients ≥ 2 samples were collected at different times of the disease (range 2-4 visits).

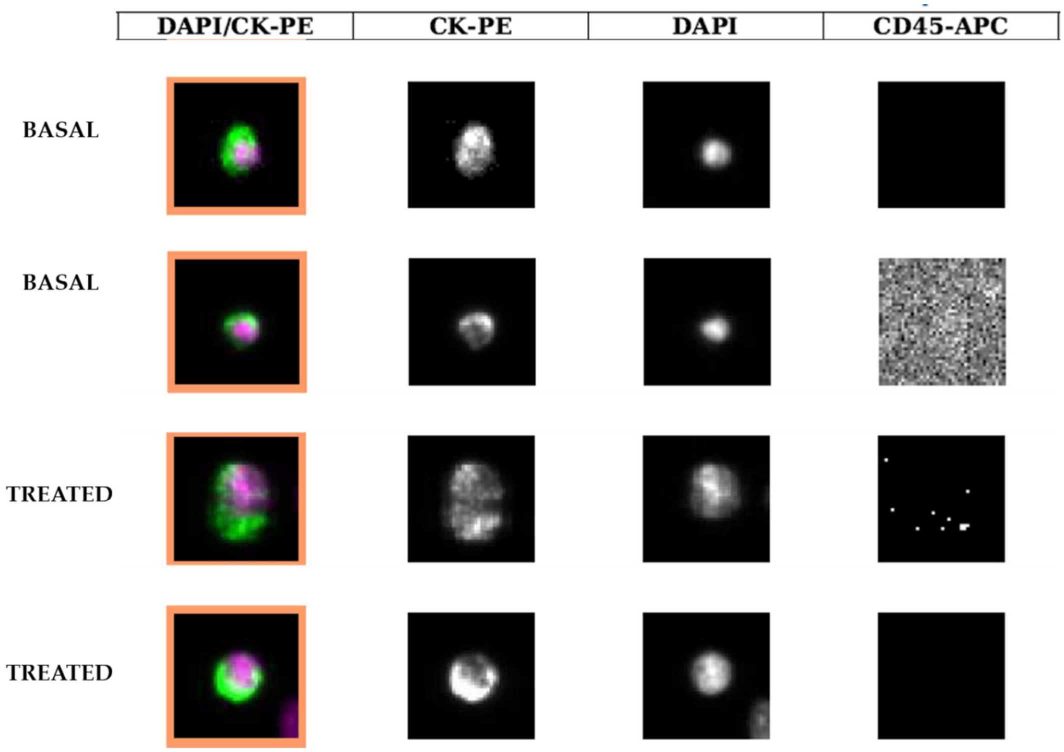

Figure S2. Representative images of CTCs isolated by the Cellsearch® system.

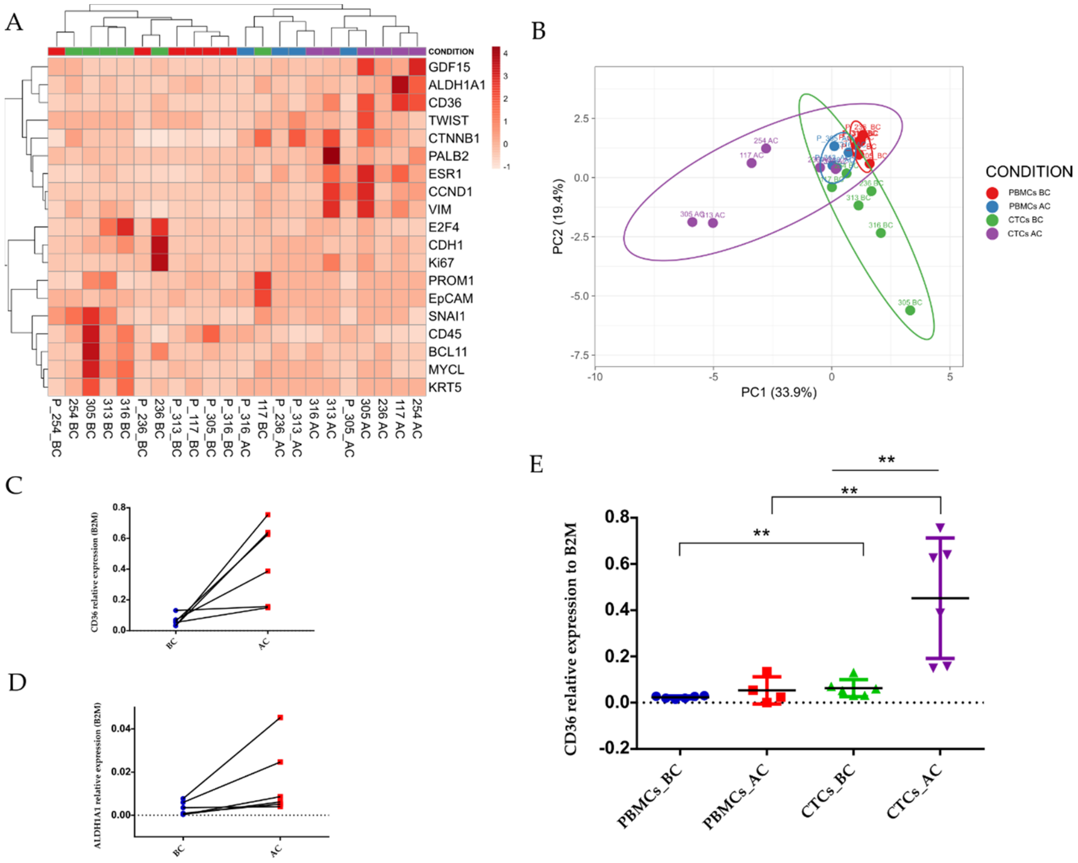

Figure S3. Gene expression analysis of PBMCs Before Culture (BC, red) and After Culture (AC, blue) and paired CTCs Before Culture (BC, green) and After culture (AC, purple) of 6 patients. PBMCs after culture we only evaluated in 4 out of 6 samples. (A) Clustered heatmap depicting CTCs and PBMCs relative gene expression (to B2M) levels of listed genes. Light red indicates no expression. (B) Principal component analysis using gene expression of listed genes (see Heatmap). CTCs after culture grouped together while CTCs before culture exhibited more similarity to PBMCs, probably due to unspecific isolated cells. (C) Relative gene expression of *CD36* gene, related to *B2M* of paired CTC samples before (BC) and after culture (AC). (D) Relative gene expression of *ALDH1A1* gene, related to *B2M* of paired CTC samples before (BC) and after culture (AC). (E) Relative gene expression of *CD36* gene, related to *B2M* for PBMCs Before Culture (BC, red) and After Culture (AC, blue) and paired CTCs before culture (BC, green) and After culture (AC, purple).

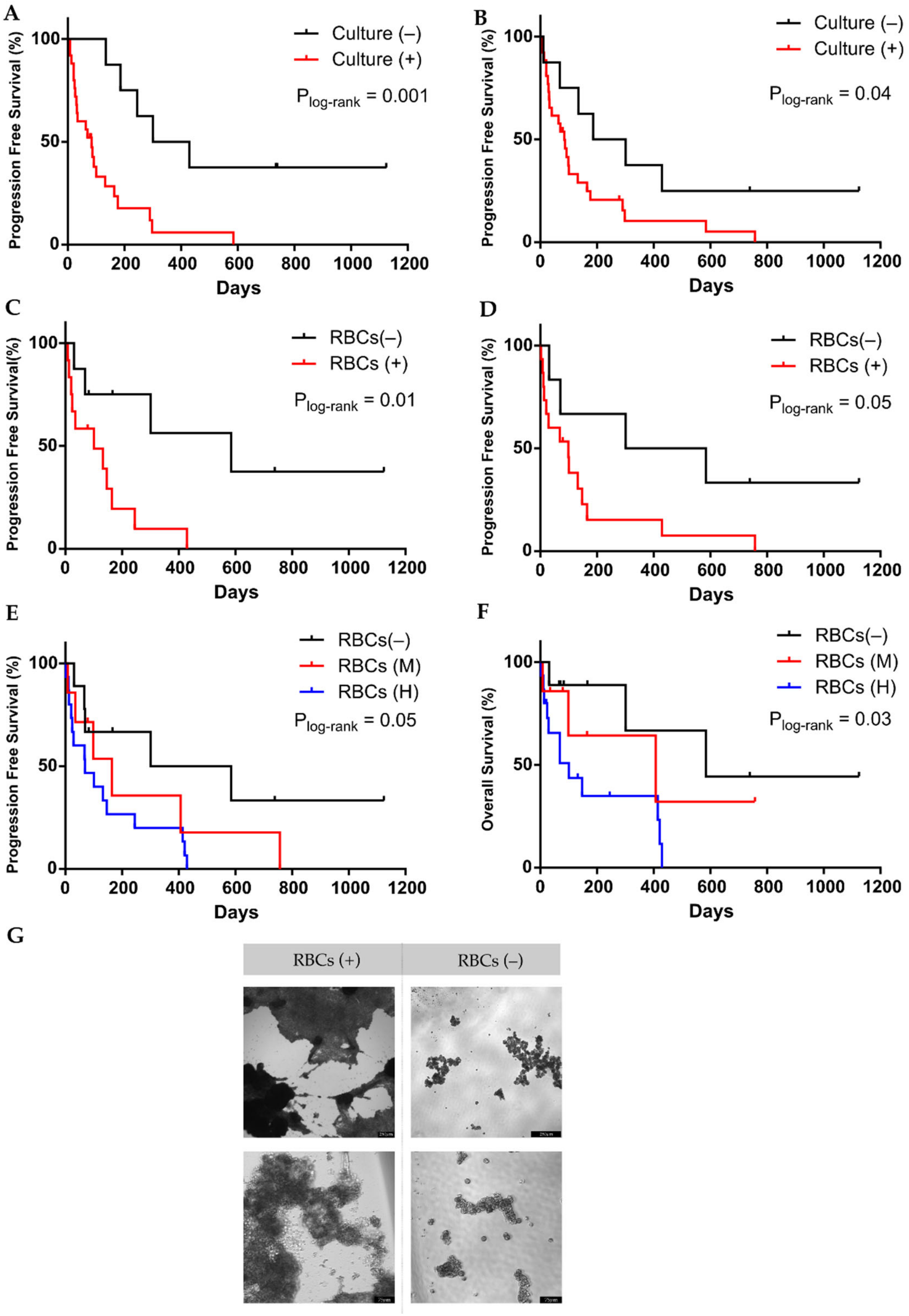

Figure S4. Survival analysis of alternative samples cohorts. A-B) Kaplan–Meier plots for PFS for cultivability (culture-negative (black) or positive (red)) in samples with paired CTC enumeration by Cellsearch® data. (A) or considering only 1 sample per patient (B); (C-D) Kaplan–Meier plots for PFS for presence (red) or absence (black) of RBCs in culture in samples with paired CTCs enumeration by Cellsearch® data (C) or considering only 1 sample per patient (D); (E-F) Kaplan–Meier plots for PFS and OS for the amount of RBCs in the culture: low or absence (black), medium (red) and high (blue). P-values were calculated using the log-rank test. (G) Representative images of RBCs in co-culture with CTCs. In the left panel, the amount of RBCs is high, forming a network among them and with the cells. In the right panel, RBCs are present in low density in the culture. .
